# Using eQTL weights to improve power for genome-wide association studies: a genetic study of childhood asthma

**DOI:** 10.3389/fgene.2013.00103

**Published:** 2013-05-31

**Authors:** Lin Li, Michael Kabesch, Emmanuelle Bouzigon, Florence Demenais, Martin Farrall, Miriam F. Moffatt, Xihong Lin, Liming Liang

**Affiliations:** ^1^Department of Biostatistics, Harvard School of Public HealthBoston, MA, USA; ^2^Department of Pediatric Pneumology and Allergy, KUNO University Children's Hospital RegensburgRegensburg, Germany; ^3^INSERM, Genetic Variation and Human Diseases Unit, U946Paris, France; ^4^Sorbonne Paris Cité, Institut Universitaire d'Hématologie, Université Paris DiderotParis, France; ^5^Wellcome Trust Centre for Human GeneticsOxford, UK; ^6^Molecular Genetics and Genomics Section, National Heart and Lung Institute, Imperial College LondonLondon, UK; ^7^Department of Epidemiology, Harvard School of Public HealthBoston, MA, USA

**Keywords:** asthma, family-wise error rate, false discovery rate, eQTL, genome-wide association study, weighted hypothesis test

## Abstract

Increasing evidence suggests that single nucleotide polymorphisms (SNPs) associated with complex traits are more likely to be expression quantitative trait loci (eQTLs). Incorporating eQTL information hence has potential to increase power of genome-wide association studies (GWAS). In this paper, we propose using eQTL weights as prior information in SNP based association tests to improve test power while maintaining control of the family-wise error rate (FWER) or the false discovery rate (FDR). We apply the proposed methods to the analysis of a GWAS for childhood asthma consisting of 1296 unrelated individuals with German ancestry. The results confirm that eQTLs are enriched for previously reported asthma SNPs. We also find that some SNPs are insignificant using procedures without eQTL weighting, but become significant using eQTL-weighted Bonferroni or Benjamini–Hochberg procedures, while controlling the same FWER or FDR level. Some of these SNPs have been reported by independent studies in recent literature. The results suggest that the eQTL-weighted procedures provide a promising approach for improving power of GWAS. We also report the results of our methods applied to the large-scale European GABRIEL consortium data.

## Introduction

Asthma is a disorder characterized by inflamed mucosa of small airways of lung, causing wheezing and shortness of breath (Moffatt et al., 2010). Among the most common chronic diseases of childhood, asthma has been reported to affect more than 10% of children in many westernized societies (Cookson, 2004). It is caused by a combination of genetic and environmental factors (Cookson, 2004; Moffatt et al., 2007), and several genome-wide association studies (GWAS) have been conducted to study the genetic basis underlying the complex disorder. More than 50 single nucleotide polymorphisms (SNPs) have been reported to be associated with asthma, according to the GWAS catalog (www.genome.gov/gwastudies, accessed on January 15, 2013). Remarkably, the recent report (Moffatt et al., 2010) from the GABRIEL (A Multidisciplinary Study to Identify the Genetic and Environmental Causes of Asthma in the European Community) consortium identified several SNPs reaching genome-wide significance through a large-scale meta-analysis.

Prior biological information, often available in practice, has potential to increase power of GWAS. The common practice of GWAS, “agnostic” in some sense, assumes no prior information about any of the SNPs under investigation, meaning that all the SNPs have an equal likelihood of being causal. Some recent studies have taken advantage of information from linkage analysis (Roeder et al., 2006) and gene expression (Xiong et al., 2012) in genome-wide association scans. In genetic studies of etiology of asthma, it is of our particular interest to employ similar approaches and explore potentials of power gain in identifying asthma-associated SNPs by incorporating expression quantitative trait loci (eQTL) information.

Catalogs of eQTLs in multiple tissues have been made publicly available, resulting from recent efforts of GWAS of gene expressions (Stranger et al., 2005, 2007, 2012; Dixon et al., 2007; Dimas et al., 2009; Yang et al., 2010). eQTLs provide insight into biology of transcription regulation. It has been shown that eQTLs are enriched for SNPs associated with complex diseases and traits using GWAS (Cookson et al., 2009; Nicolae et al., 2010). eQTL results can be used to provide functional interpretation for findings from GWAS (Moffatt et al., 2007; Heid et al., 2010; Hsu et al., 2010; Lango Allen et al., 2010; Speliotes et al., 2010; Chu et al., 2011; Wu et al., 2012) and prioritize genes in an association region for carrying out functional experiments using animal models (Teslovich et al., 2010). Focusing on eQTLs may also be useful to identify genetic pathways associated with the risk of complex diseases and traits, such as basal cell carcinoma in a skin cancer GWAS (Zhang et al., 2012) and type 2 diabetes (Zhong et al., 2010). Other results show that many *cis* eQTLs are shared across tissues (Ding et al., 2010) and that a comprehensive eQTL catalog in one tissue might be used to increase the power of capturing relevant transcripts for other diseases (including those that are only weakly or incidentally expressed in tissues where eQTL information was collected).

As single-SNP analysis still remains the most popular in GWAS, we focus on those methods designed for this type of analysis. Single-SNP analysis tests one SNP at a time for association by scanning across the whole genome, and hence involves a large number of hypotheses. To correct for multiple comparisons, statistical methods have been proposed and applied to control for the family-wise error rate (FWER) (Bonferroni, 1936; Holm, 1979) or the false discovery rate (FDR) (Benjamini and Hochberg, 1995; Storey and Tibshirani, 2003). Recent advances in statistical methodology make it possible to incorporate prior information through weighted hypothesis testing. In several of such methods (Genovese et al., 2006; Roeder et al., 2006, 2007), hypotheses are up-weighted or down-weighted based on prior likelihood of association with the trait of interest. While keeping the FWER or FDR under control, the procedures can improve power with informative weights and suffer small loss in power with uninformative weights (Genovese et al., 2006; Roeder and Wasserman, 2009). This feature is appealing as compared to prescreening SNPs based on prior information (e.g., to consider only eQTLs for association testing). In this paper, we propose to use eQTLs as prior information, and apply these weighted hypothesis testing methods to reanalyze the MAGICS (Multicentre Asthma Genetics in Childhood Study) data of asthma GWAS (Moffatt et al., 2007) as well as the GABRIEL meta-study of asthma (Moffatt et al., 2010).

## Results

### Published asthma associations are enriched with eQTLs

We extracted published asthma associations from the GWAS catalog maintained by the National Human Genome Research Institute. As of January 15, 2013, 52 distinct reference SNPs in or near more than 40 genes have been reported to be associated with asthma (Table A1). According to the eQTL database (described in Materials and Methods), 20 of these 52 SNPs (38.5%) are eQTLs. Using the proxy SNP search tool SNAP (Johnson et al., 2008), we then obtained an extended list of 506 SNPs that were either in the GWAS catalog or in strong linkage disequilibrium (LD) with the 52 SNPs (*r*^2^≥ 0.8). We called all these 506 SNPs the extended set of asthma-associated SNPs.

We calculated an eQTL enrichment *p*-value (Hosack et al., 2003) using the MAGICS data. There are 300,821 SNPs that passed quality control in the MAGICS data. Among these SNPs, 29 SNPs are in the GWAS catalog, and 64 SNPs are among the 506 extended asthma-associated SNPs defined previously. To account for the LD between SNPs in the calculation of enrichment *p*-value, we conducted LD pruning with the *r*^2^ threshold of 0.8 on the 300,821 SNPs. This resulted in 251,826 SNPs and 38 of them are extended asthma-associated SNPs according to the GWAS catalog. According to the eQTL database, 22,922 SNPs (9.1% of 251,826 SNPs) are eQTLs, and 13 asthma associated SNPs (34.2% of 38 SNPs) are eQTLs. The corresponding enrichment *p*-value is 6.78 × 10^−5^, suggesting the asthma associations are enriched with eQTLs in the MAGICS data. Note that other analyses considered all the SNPs rather than the pruned set of SNPs.

These results are in line with the previous findings (Nicolae et al., 2010), which studied the eQTLs in lymphoblastoid cell lines (LCL) from the HapMap samples and the GWAS catalog. Their results suggest that SNPs associated with complex traits are more likely to be eQTLs.

### Weights using eQTL information

**Figure 1:**
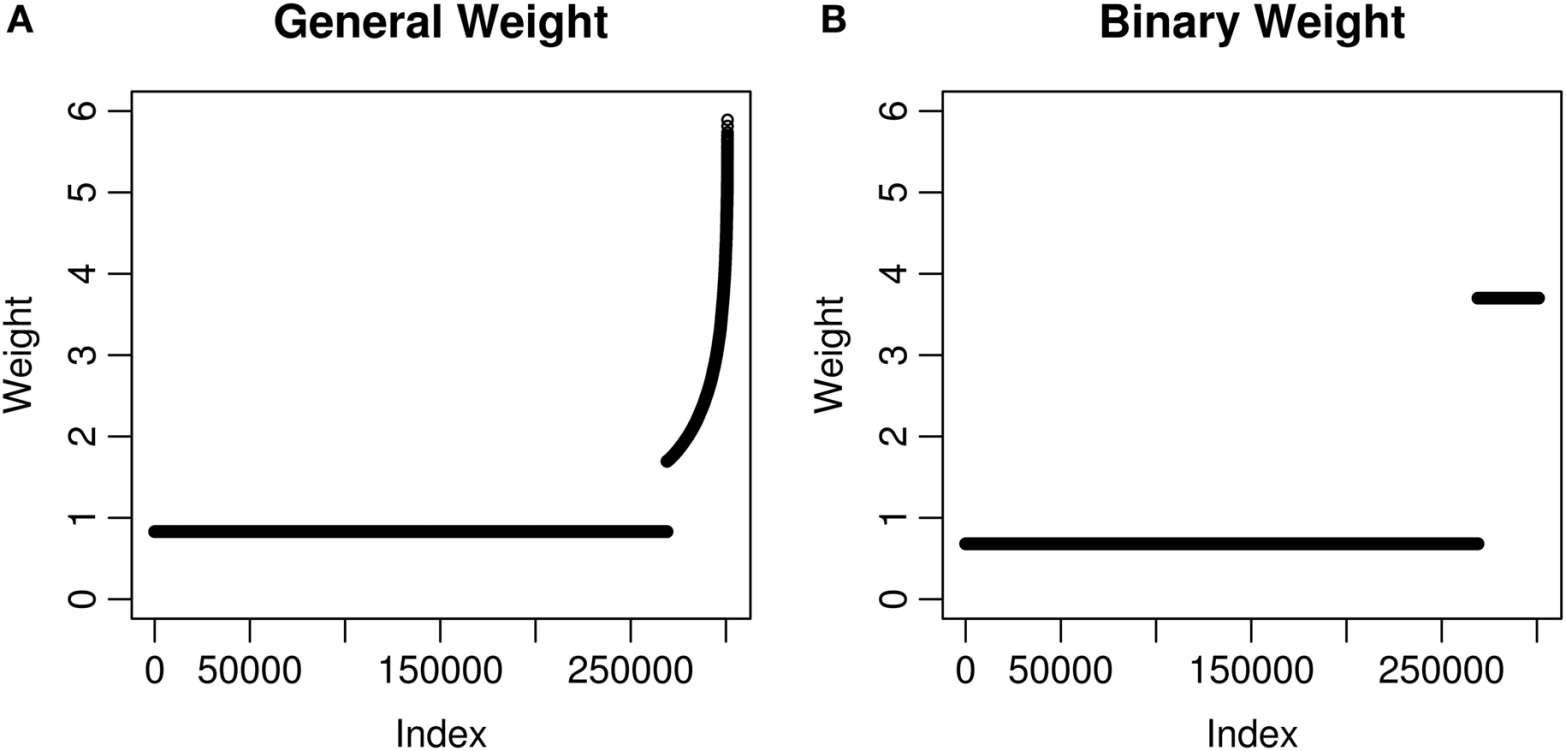
**Weights used in the MAGICS analysis.** Each weight corresponds to a SNP and a hypothesis. The weights have been normalized to have mean 1 and shown in the ascending order. **(A)** The weights are based on the square root of −log_10_ *p*_eQTL_ where *p*_eQTL_ is the eQTL *p*-value; **(B)** the weights take only two possible values, which are decided using the method described in Materials and Methods.

We calculated two kinds of weights using eQTL information for the MAGICS data. All the 300,821 SNPs passing quality control were considered. First, we defined a SNP as an eQTL SNP if it was labeled as an eQTL in the eQTL database (see details in Materials and Methods). There are 31,781 *cis* eQTL SNPs (10.6% of 300,821 SNPs) according to the definition, and for each of them, we retrieved an eQTL *p*-value *p*_eQTL_. Next, we considered two choices of weights, the general weight and the binary weight. The general weight is $w_{g}=\sqrt{-\log_{\text{10}}p_{\text{ eQTL}}}$ for an eQTL SNP, and *w*_g_ = 1 otherwise. The binary weight takes only two possible values, *w*_*b*_ = 3.70 for any eQTL SNP and *w*_*b*_ = 0.68 otherwise. The two values of the binary weight were chosen to maximize the minimum power while keeping at least 10.6% (also the percentage of eQTL SNPs) of all the hypotheses with a power of 60%. The parameters for calculating the binary weight are ϵ = 0.106, α = 0.05, and β = 0.4 (see details in Materials and Methods). Last, both weights were normalized to have the mean equal to 1 which is necessary for the weighted hypothesis testing methods to maintain the correct FWER or FDR (Genovese et al., 2006). After normalization, the general weight *w*_*g*_ has a mean of 2.44 and a median of 2.21 among eQTL SNPs, while the binary weight *w*_*b*_ is 3.70 for all eQTL SNPs (Figure 1).

### Weighted hypothesis testing

We applied the weighted hypothesis testing methods (Genovese et al., 2006; Roeder and Wasserman, 2009) using the general weight *w*_*g*_ and the binary weight *w*_*b*_ to the MAGICS data. For each of the 300,821 SNPs, we calculated the trait association *p*-value, *p*, from the single-SNP association test on the phenotypes of asthma status, as well as the weighted *p*-values *Q*_*g*_ = *p*/*w*_*g*_ and *Q*_*b*_ = *p*/*w*_*b*_. Multiple testing adjustments were done for both the original *p*-values (*p*) and the weighted *p*-values (*Q*_*g*_ and *Q*_*b*_). Bonferroni (1936) and Holm's (1979) methods were considered to control for FWER, and Benjamini and Hochberg's (1995) method was used to control for FDR.

**Figure 2:**
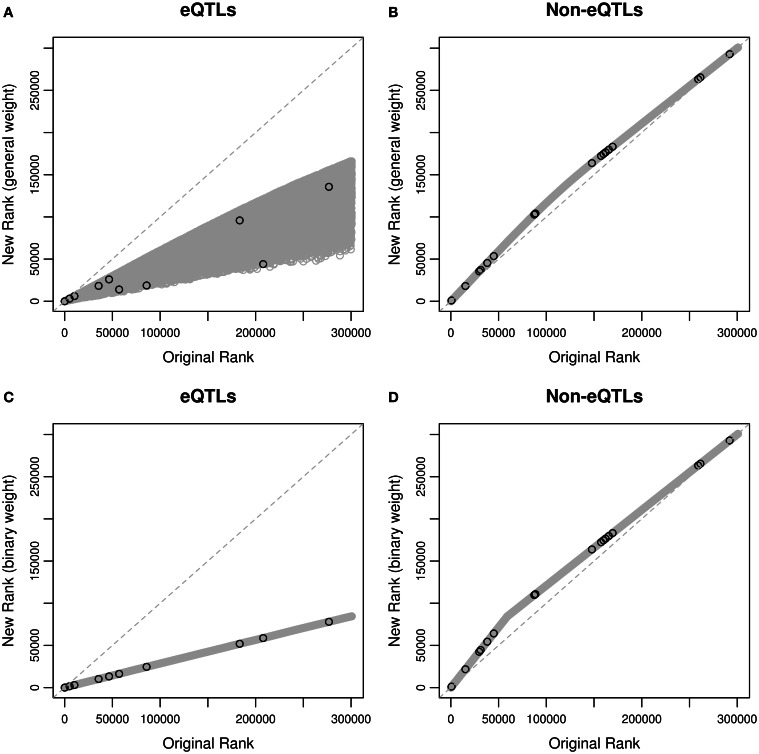
**Rankings of the SNPs based on original *p*-values and weighted *p*-values in the MAGICS analysis. (A)** Original ranks of eQTLs compared to their new ranks based on the general weight; **(B)** original ranks of non-eQTLs compared to their new ranks based on the general weight; **(C)** original ranks of eQTLs compared to their new ranks based on the binary weight; **(D)** original ranks of non-eQTLs compared to their new ranks based on the binary weight. The black circles represent the reported asthma-associated SNPs in the GWAS catalog, and the gray circles represent the rest of the SNPs in the data.

We first ranked the SNPs using their *p*-values in the ascending order, and compared the ranks based on the weighted *p*-values with those based on the original *p*-values (Figure 2). Since only 10.6% SNPs are eQTL SNPs according to the eQTL database, and hypotheses for eQTL SNPs are up-weighted, eQTLs generally have higher ranks after weighting, and non-eQTLs' ranks are lower but the magnitude of changes is small. This is true for both the general and binary weights. When restricting to the 29 asthma-associated SNPs reported in the GWAS catalog, we also observed similar behaviors, suggesting that weighting hypotheses may improve power using informative weights, and sacrifice a little power using uninformative weights (Roeder and Wasserman, 2009).

**Figure 3:**
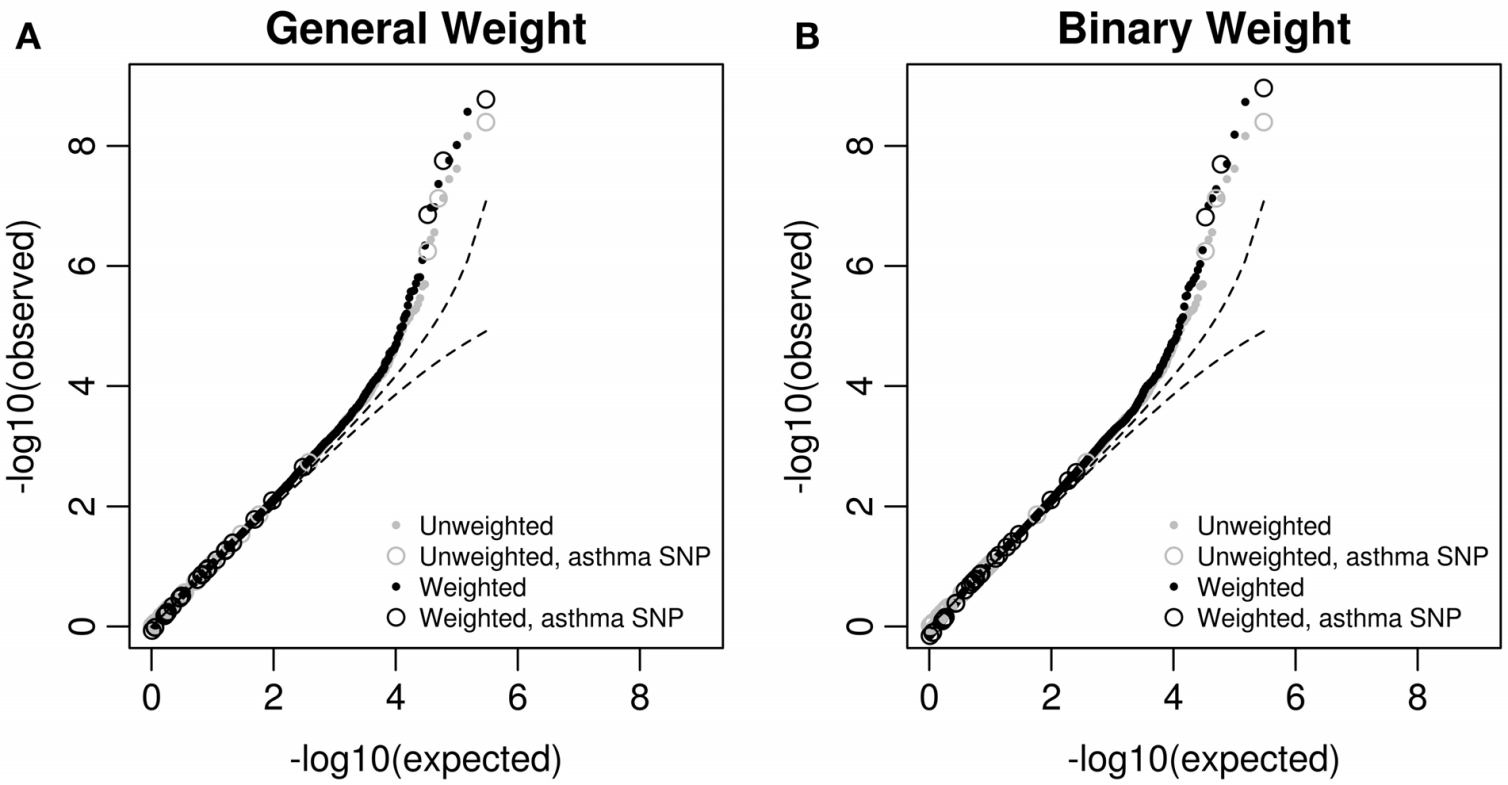
**Q–Q plots of original *p*-values and weighted *p*-values in the MAGICS analysis.** The weighted *p*-values are based on **(A)** the general weight, or **(B)** the binary weight. The reported asthma-associated SNPs in the GWAS catalog are shown in circles.

We then looked at the Q–Q plots of the original and weighted *p*-values. For *p*-values greater than 0.0001, the Q–Q curves (Figure 3) are similar between the original and weighted *p*-values, regardless of the weights used. For those *p*-values less than 0.0001, some weighted *p*-values are smaller than original ones, and the difference is larger using the binary weight. We also observed that 3 asthma-associated SNPs in the GWAS catalog are among the top SNPs with original *p*-values less than 10^−6^. The weighted *p*-values for all the 3 asthma-associated SNPs are smaller than original ones.

Next, we applied the methods to control for the FWER. An effective ratio of 0.791 (Li et al., 2012) was used to calculate the effective number of SNPs (300,821 × 0.791). Controlling for an FWER level of 0.05, we obtained significant SNPs using both the original and weighted *p*-values. Both Bonferroni and Holm's methods gave the same results, and both weights (binary and general) also gave the same results (Tables A2, A3). The unweighted hypothesis testing claimed 6 SNPs to be significant, all on chromosome 17, including 2 asthma-associated SNPs (rs3894194 with *GSDMA*, and rs7216389 with *ORDML3*) that have been reported previously (Moffatt et al., 2007, 2010). After applying the weighted hypothesis testing, we obtained 9 significant SNPs including all the 6 SNPs identified by the unweighted method, although the ranks are not exactly the same. The 3 SNPs additionally identified by eQTL weighting were rs3902025, rs4795405, and rs2305480. The SNP rs2305480, a missense SNP in the gene *GSDMB*, was not reported in the previous GWAS study (Moffatt et al., 2007) but has been reported as an asthma-associated SNP in a later larger scale study by the GABRIEL consortium (including the MAGIC data, Moffatt et al., 2010) and was found to be strongly interacting with exposure to tobacco smoke in early life (Bouzigon et al., 2008). We found that rs2305480 is actually in LD (*r*^2^ = 0.702, *D*′ = 0.926) with rs7216389 that was identified by the unweighted methods, suggesting that rs2305480 may not represent a new association. Using a stringent *r*^2^ threshold of 0.4, we found that the other two SNPs, rs3902025 and rs4795405, are also in LD with at least one SNP identified by the unweighted methods. So there is no new association identified by the weighted methods in this particular analysis.

Besides controlling for FWER, we also used Benjamini and Hochberg's (BH) procedure (Benjamini and Hochberg, 1995) to control for a FDR level of 0.05 (Table A4). Based on the original *p*-values without weighting, the BH procedure gave 11 positive results (SNPs). The weighted BH procedures based on the general weight and the binary weight resulted in 7 and 8 additional positive results (SNPs), respectively. Using a stringent *r*^2^ threshold of 0.4, we found that 5 SNPs (Table 1) are not in LD with any of the SNPs identified without weighting. Although none of the 5 SNPs are, or in LD with, any asthma-associated SNPs according to the GWAS catalog, there are some SNPs that seem interesting. Some of the SNPs are in or close to the genes *PGAP3* and *STARD3* on chromosome 17, and interestingly, rs2941504 has been reported in a recent independent study (Anantharaman et al., 2011) to be associated with asthma, although it does not meet the criteria for inclusion in the GWAS catalog. This suggests that the reanalysis using the eQTL weighting approaches is promising and potentially useful.

**Table 1:** **Additional significant SNPs or positive results identified by eQTL weighting methods after accounting for linkage disequilibrium in the MAGICS analysis**.

**Chr**	**SNP**	**Gene**	***p*-value**	***Q*_*g*_**	***Q*_*b*_**	**Method**
17	rs1877031	*STARD3*	4.32 × 10^−6^	1.54 × 10^−6^	1.17 × 10^−6^	BH
17	rs931992	*TCAP, STARD3*	5.61 × 10^−6^	1.96 × 10^−6^	1.52 × 10^−6^	BH
17	rs1565922	*PGAP3*	7.28 × 10^−6^	2.55 × 10^−6^	1.97 × 10^−6^	BH
17	rs2941504	*PGAP3*	7.64 × 10^−6^	2.68 × 10^−6^	2.06 × 10^−6^	BH
10	rs11191325	*SUFU*	8.49 × 10^−6^	3.37 × 10^−6^	2.29 × 10^−6^	BH

**Q*_*g*_ and *Q*_*b*_ are the eQTL weighted p-values based on the general weight and the binary weight, respectively. Using both weights result in the same set of SNPs*.

### Reanalysis of the GABRIEL data

As another application, we reanalyzed the GABRIEL data using the eQTL weighted approaches. Since only the *p*-values are necessary for the use of eQTL weighting, we took the *p*-values of the meta analysis of 37 studies that were calculated based on imputed data. In total, there are 2,473,850 SNPs and their *p*-values available in the GABRIEL study, which include 267,350 out of 268,204 eQTL SNPs in the eQTL database. The weights based on eQTL information were calculated in the similar way to the MAGICS data analysis.

We applied Bonferroni and Holm's methods with an FWER level of 0.05, as well as the BH procedure with an FDR level of 0.05. An effective ratio of 0.30 (Li et al., 2012) was used to calculate the effective number of SNPs (2,473,850 × 0.30). After obtaining the lists of significant SNPs using different methods, we report any SNPs identified by eQTL weighting that are not in LD with any SNPs identified by unweighted methods using an *r*^2^ threshold of 0.4. Such SNPs may be informative and suggest new associations. Tables 2 and 3 show the SNPs that were identified based on the general weight and the binary weight, respectively.

**Table 2:** **Additional significant SNPs or positive results identified by eQTL weighting methods after accounting for linkage disequilibrium in the GABRIEL analysis**.

**Chr**	**SNP**	**Gene**	***p*-value**	***Q*_*g*_**	***Q*_*b*_**	**Method**
5	rs244749	*MEF2C*	6.25 × 10^−5^	2.51 × 10^−5^	1.54 × 10^−5^	BH
5	rs10075941		6.13 × 10^−5^	3.03 × 10^−5^	1.51 × 10^−5^	BH
17	rs7503195		7.66 × 10^−5^	3.07 × 10^−5^	1.88 × 10^−5^	BH
17	rs17637472		6.13 × 10^−5^	3.56 × 10^−5^	1.51 × 10^−5^	BH
9	rs7047575		6.87 × 10^−5^	3.92 × 10^−5^	1.69 × 10^−5^	BH

*The results are based on the general weight*.

**Table 3:** **Additional significant SNPs or positive results identified by eQTL weighting methods after accounting for linkage disequilibrium in the GABRIEL analysis**.

**Chr**	**SNP**	**Gene**	***p*-value**	***Q*_*g*_**	***Q*_*b*_**	**Method**
5	rs736801		2.15 × 10^−7^	8.81 × 10^−8^	5.29 × 10^−8^	Bonferroni, Holm
6	rs2596450	*HCG26*	2.72 × 10^−7^	1.16 × 10^−7^	6.69 × 10^−8^	Bonferroni, Holm
5	rs10075941		6.13 × 10^−5^	3.03 × 10^−5^	1.51 × 10^−5^	BH
17	rs17637472		6.13 × 10^−5^	3.56 × 10^−5^	1.51 × 10^−5^	BH
5	rs244749	*MEF2C*	6.25 × 10^−5^	2.51 × 10^−5^	1.54 × 10^−5^	BH
9	rs7047575		6.87 × 10^−5^	3.92 × 10^−5^	1.69 × 10^−5^	BH
17	rs7503195		7.66 × 10^−5^	3.07 × 10^−5^	1.88 × 10^−5^	BH
6	rs9273363	*HLA_DQB1*	8.38 × 10^−5^	4.41 × 10^−5^	2.06 × 10^−5^	BH
5	rs4351182		9.98 × 10^−5^	4.91 × 10^−5^	2.45 × 10^−5^	BH
2	rs13391794		1.01 × 10^−4^	5.62 × 10^−5^	2.49 × 10^−5^	BH
5	rs10044342	*MEF2C*	1.10 × 10^−4^	4.59 × 10^−5^	2.71 × 10^−5^	BH
2	rs6751196		1.14 × 10^−4^	6.22 × 10^−5^	2.80 × 10^−5^	BH
6	rs176095	*PBX2*, *GPSM3*	1.25 × 10^−4^	5.12 × 10^−5^	3.08 × 10^−5^	BH
2	rs2675073		1.34 × 10^−4^	5.40 × 10^−5^	3.29 × 10^−5^	BH
2	rs1913621		1.46 × 10^−4^	7.92 × 10^−5^	3.60 × 10^−5^	BH
2	rs10497621	*NUP35*	1.59 × 10^−4^	7.55 × 10^−5^	3.91 × 10^−5^	BH
5	rs244750	*MEF2C*	1.70 × 10^−4^	7.08 × 10^−5^	4.17 × 10^−5^	BH
6	rs9366689	*POM121L2*	1.72 × 10^−4^	8.73 × 10^−5^	4.23 × 10^−5^	BH
6	rs7775759		1.81 × 10^−4^	6.43 × 10^−5^	4.46 × 10^−5^	BH
6	rs7741091		1.81 × 10^−4^	6.43 × 10^−5^	4.46 × 10^−5^	BH
8	rs6601649		1.90 × 10^−4^	9.68 × 10^−5^	4.68 × 10^−5^	BH
6	rs204994		1.94 × 10^−4^	8.02 × 10^−5^	4.76 × 10^−5^	BH

*The results are based on the binary weight*.

### Size simulations on FWER

We conducted simulations using 5000 permutations based on the MAGICS data, and calculated the percentage of having at least one false positive claimed by Bonferroni and Holm's methods (α = 0.05, with an effective ratio of 0.791). In fact, any SNPs claimed significant using the two methods would be a false positive. The calculated percentages (Table 4) provide estimates of the FWER. Bonferroni and Holm's methods give the same results. The results suggest that, under the null hypothesis, the FWER level is controlled for the methods based on both the original (unweighted) and the weighted *p*-values. The simulations confirm the validity of the weighted hypothesis method (Genovese et al., 2006).

**Table 4:** **Family-wise error rate estimates in 5000 permutations**.

***P*-value**	**FWER**
Original	0.0454
Weighted by general weight	0.0458
Weighted by binary weight	0.0460

*Both Bonferroni and Holm's methods gave the same results in the same scenarios*.

## Discussion

It is of substantial interest to enhance the power for identifying associations in the era of post-GWAS. Besides meta-analysis that has been proved successful in power gain (Moffatt et al., 2010), incorporating prior information has also received increasing attention. Such information can be obtained from various sources and levels, such as linkage analysis (Roeder et al., 2006), gene expression (Yang et al., 2010), and annotation information of variants (Adzhubei et al., 2010), genes (Saccone et al., 2007), and pathways (Wang et al., 2007). The so-called “agnostic” GWAS may benefit from incorporating useful prior information. In our study of asthma, gene expression information is of particular interest, as a recent study (Moffatt et al., 2007) identified several eQTLs associated with asthma.

In the reanalysis of the MAGICS data (Moffatt et al., 2007), we applied recently developed statistical methods that can improve power by weighting hypothesis (Genovese et al., 2006; Roeder and Wasserman, 2009). Using eQTL information obtained from an independent dataset, we employed weighted procedures that up-weighted eQTL SNPs and down-weighted non-eQTL SNPs while controlling for the FWER or the FDR. It has been proved (Genovese et al., 2006) that any set of nonnegative weights can guarantee substantial power gain given informative weights and little power loss for uninformative weights. The property implies that the weighted procedures are robust to informativeness of weights and to the uneven coverage of genes and expression targets on the genome. We took advantage of this robustness and applied the procedures to an asthma study. We found additional SNPs that were significantly associated with asthma according to the weighting hypothesis methods. Some of them were interesting after we accounted for LD and compared them to literature. Our analysis was the first application of this approach to asthma GWAS studies, and the results successfully illustrated the use of eQTL weighting in the context of asthma studies. As another application, we also reanalyzed the GABRIEL meta-analysis *p*-values and reported corresponding results.

It is noted that the weighted procedures can utilize eQTL information from a reference database. Multiple choices of eQTL databases have already been made available (e.g., Yang et al., 2010; Liang et al., 2013), and future efforts may provide even better reference of eQTL information. For example, the eQTL information considered in our reanalysis was obtained through a single platform (Affymetrix HG-U133 Plus 2.0), and better coverage of gene expression profiling may be achieved through RNA-Seq technologies or by combining information from various platforms.

Besides the weighted procedures, an alternative method of using eQTL information is to simply test association between eQTL SNPs and the trait of interest. Such a method is not recommended in GWAS as it excludes non-eQTL completely and relies on the prior information too heavily. By contrast, weighted procedures make it possible to consider eQTLs and non-eQTLs simultaneously. More importantly, they can possibly increase power if the prior information is useful and are able to maintain the type I error under the null.

Applying the weighted procedures in our reanalysis only requires *p*-value of eQTL SNPs. This flexibility means that such analyses can be applied to any existing GWAS data, even if they do not have accompanying gene expression data. Although gene expression may have tissue-specific patterns, a substantial fraction of eQTLs may be shared across tissues (Ding et al., 2010). Hence eQTLs developed from tissues that are not directly relevant to the outcome of interest, such as those from publicly available eQTL databases based on LCL, can be used to improve power on GWAS. It is possible that using eQTL information from relevant tissues may result in even more power gain, if such information is available.

Besides the particular weighting hypothesis method (Genovese et al., 2006) we adopted, Bayesian methods are potentially alternative strategies to incorporate eQTL information. The use of Bayes factors has been applied to genetic association studies (Wellcome Trust Case Control Consortium, 2007; Stephens and Balding, 2009). In single SNP analysis, a prior is assumed for each SNP effect [e.g., *N*(0, 0.2^2^) under a model of association in Wellcome Trust Case Control Consortium (2007)]. eQTL information can be naturally incorporated into the prior, although it may be challenging to choose a realistic yet tractable alternative model and to assess error rates (Hoggart et al., 2008), especially with the eQTL weight. One possible choice is through modifying the variance of the prior, for example assuming a prior *N*(0.2^2^*w*), where *w* is a weight of eQTL signal. Another possible choice is to keep the variance the same and increase the probability of association for eQTLs *a priori*. It is of interest in future research to explore these possibilities and consider the extension of Bayesian methods to incorporate eQTL information.

In our analysis, we took into account the LD between SNPs by considering the effective number of SNPs (Li et al., 2012). As an alternative, testing SNP sets for association has potential of improving power and reducing the correlation between tests. Since the focus of this paper is to demonstrate the use of eQTL information in association testing, we will consider the weighted correlated hypothesis in future research.

Two choices of weights were applied in our analysis including a binary weight and a weight using strength of eQTLs, and the results using the two weights were similar in our analysis. Theoretical results exist (Roeder and Wasserman, 2009) for the optimal binary weight, which provide guidance in choosing the values of the weight. The weight taking advantage of the eQTL strength may possibly provide more useful information, and what is the best choice of weights is still under research.

Through an application to an asthma GWAS, we demonstrated the usefulness of eQTL weights in GWAS. Although results may vary depending on the traits of interest and the underlying biological mechanism, the potentials of increasing power and little investment required for reanalysis make the eQTL-weighted procedures desirable for reanalysis of existing GWAS data and useful for design and analysis of future studies.

## Materials and methods

### The MAGICS asthma GWAS samples and data

The MAGICS (Multicentre Asthma Genetics in Childhood Study) study data (Moffatt et al., 2007), part of the GABRIEL consortium, were reanalyzed by incorporating eQTL information. Quality control procedures were conducted similarly to a published protocol (Anderson et al., 2010). Individuals with missing phenotypes, elevated missing rates (≥ 5%), or outlying heterozygosity rate were removed. Markers with an excessive missing rate (≥ 5%), low MAF (<5%), or failing in the HWE test (*p*-value < 10^−5^) were all excluded as well. The remaining dataset contains 1296 individuals (647 affected and 649 unaffected) genotyped across 300,821 SNPs.

**Figure A1:**
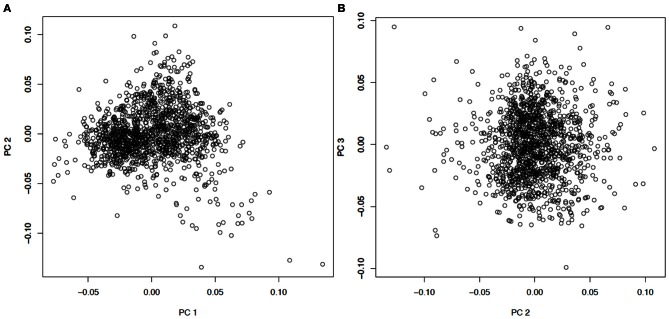
**Principal components analysis for the MAGICS study.** The scatter plots of **(A)** PC1 vs. PC2, and **(B)** PC2 vs. PC3 are shown, with each circle corresponding to an individual in the asthma GWAS.

To account for possible divergent ancestry and population stratification, principal component analysis (PCA) was conducted using EIGENSOFT 4.2 (Patterson et al., 2006; Price et al., 2006). The genotype data were pruned for LD prior to the PCA. The PCA result (Figure A1) suggests that no obvious stratification exists, and the signal of the first principal component is very weak. In the subsequent analysis, we still included the first principal component as a covariate.

LD pruning was considered only in the calculation of enrichment *p*-value. It was conducted using PLINK (v1.07, downloaded from http://pngu.mgh.harvard.edu/purcell/plink/) (Purcell et al., 2007). A moving window with a width of 50 SNPs and a step size of 5 SNPs was considered, and pairwise LDs were calculated and pruned if *r*^2^ > 0.8 (corresponding PLINK arguments: “–indep-pairwise 50 5 0.8”).

### The GABRIEL meta-analysis *p*-values

Association testing results, including SNP ID and *p*-values, were obtain from a reanalysis of the GABRIEL consortium data using imputed SNPs (Bouzigon et al., personal communication). The meta-analysis considered imputation of SNP genotypes using the HapMap 2 reference data for 37 studies, and calculated a meta-analysis *p*-value for each SNP using available data. Imputed SNPs were kept for analysis if their imputation scores (Rsq) were ≥ 0.5 and if their minor allele frequencies were ≥ 1%. In total there were 2,473,850 SNPs that passed the quality control. Only the SNP ID and the *p*-values of these SNPs were obtained and used for the reanalysis described in this paper.

### Expression quantitative trait loci data

An eQTL database (http://www.hsph.harvard.edu/liming-liang/software/eqtl/) resulting from an independent dataset was used as prior information to be incorporated in the GWAS. The sample contains 405 siblings from a panel of families of British descent (MRC-A) (Dixon et al., 2007). Global gene expression in LCLs was measured using Affymetrix HG-U133 Plus 2.0 chips. All siblings were genotyped using the Illumina Sentrix HumanHap300 BeadChip (ILMN300K) and/or the Illumina Sentrix Human-1 Genotyping BeadChip (ILMN100K). The SNP genotype data were further imputed using the MaCH program, and each SNP was tested for association with probes in the gene expression data. Restricting to *cis* eQTLs (1 Mb region) and controlling for the FDR of 1%, there are 515,947 tests with logarithm of odds (LOD) scores greater than 3.172, corresponding to 268,204 unique SNPs. In case a SNP has multiple *p*-values reported for associations with different probes, the minimal *p*-value was used for that SNP. These 268,204 SNPs are considered as eQTLs, and the database contains information of their physical positions, LOD scores, *p*-values, and residing or nearby genes. Details of the database are described by Liang et al. (2013).

### Genetic association analysis

Genetic association analysis of the MAGICS data was conducted in PLINK. Logistic regression was used to test for disease-trait SNP association while adjusted for gender and the first principal component. Meta-analysis on GABRIEL data was carried out by combining association results from 37 studies using a random effect model, and all computations were done using Stata software.

### *p*-value weighting methods

Consider *m* hypotheses *H*_1_, …, *H*_*m*_ and their test statistic *p*-values, *P*_1_, …, *P*_*m*_. Suppose there are weights *W*_1_, …, *W*_*m*_ available for the *m* tests, respectively, satisfying *W*_*i*_ > 0 and ∑^*m*^_*i* = 1_*W*_*i*_ = *m*. Define *Q*_*i*_ = *P*_*i*_/*W*_*i*_ and let *Q*_(1)_ ≤ … ≤ *Q*_(*m*)_ be the sorted values. Let *P*_(1)_, …, *P*_(*m*)_ and *W*_(1)_, …, *W*_(*m*)_ be the values in the corresponding order. *Q*_*i*_ is sometimes referred to a “weighted *p*-value” (e.g., Roeder and Wasserman, 2009), although it is not a *p*-value.

The weighted Bonferroni procedure is to reject any hypothesis *H*_*j*_ (1 ≤ *j* ≤ *m*) that satisfies *Q*_*j*_ ≤ α/*m*, where α is the desired level of FWER. Genovese et al. (2006) showed that this procedure controls FWER at level no greater than α.

Holm's weighted procedure (1979) is carried out as follows: given the desired α level of FWER, if *Q*_(1)_ ≥ α/*m*, no hypothesis is rejected; otherwise, find the largest *j* that satisfies *Q*_(*i*)_ ≤ α/∑^*m*^_*k* = *i*_*W*_(*k*)_ for all *i* ≤ *j*, and reject the hypotheses corresponding to the *j* smallest *Q*_*j*_'s. Genovese et al. (2006) also prove that this procedure can work for a general setting of weights.

We also consider Benjamini and Hochberg's procedure (1995) for controlling FDR. Given the desired level α, find the largest *j* such that *Q*_(*j*)_ ≤ α · *j*/*m*, and reject the hypotheses corresponding to the *j* smallest *Q*_*j*_'s. Genovese et al. (2006) prove that this procedure controls FDR at level α.

### eQTL information as weights

The eQTL *p*-values were used to construct weights for the SNPs in the asthma GWAS reanalysis. We considered two kinds of weights, the binary weight *w*_*b*_ and the general weight *w*_*g*_. The binary weight takes only two possible values that are predefined, denoted by *w*_eQTL_ and *w*_non−eQTL_. For *m* hypotheses, a binary weight is defined as *w*_*b*_ = (*w*_*b*, 1_, …, *w*_*b*, *m*_) where *w*_*b*, *j*_ = *w*_eQTL_ if the *j*th SNP is an eQTL SNP, and *w*_*b*, *j*_ = *w*_non−eQTL_ if it is not an eQTL SNP. Given the values of α, β, and ϵ, the optimal values of *w*_eQTL_ and *w*_non−eQTL_ were chosen (Roeder and Wasserman, 2009) to maximize the minimum power among all the hypotheses while having at least a fraction ϵ with high power 1−β. Here α is either the level of FWER or FDR. We also considered a general weight, where the weight *w*_*g*_ = (*w*_*g*, 1_, …, *w*_*g*, *m*_) has $w_{g,\;j}=\sqrt{-\log_{10}p_{\text{eQTL}}}$ if the *j*th SNP is an eQTL SNP with the eQTL *p*-value *p*_eQTL_, and *w*_*g*, *j*_ = 1 otherwise. The particular form was intuitively chosen prior to the reanalysis of the GWAS data in consideration of avoiding up-weighting top eQTL SNPs too much. Both *w*_*b*_ and *w*_*g*_ were then normalized such that the means equal to 1, i.e., ${\bar{w}}_{B}=1$ and ${\bar{w}}_{G}=1$.

### Reported associations in the GWAS catalog

Asthma-associated SNPs and genes reported in publications were retrieved from the online catalog of published GWAS on January 15, 2013. The catalog limits the associations to those with *p*-values less than 1.0 × 10^−5^ and records only one SNP with a gene or region of high LD unless there was evidence of independent association. The reported associations were compared against the findings in the asthma GWAS data we reanalyzed.

### Linkage disequilibrium information

To account for LD between SNPs, LD information based on HapMap 2 was obtained. The SNAP proxy search tool (http://www.broadinstitute.org/mpg/snap/ldsearch.php) was used to obtain the information, based on the HapMap 2 (rel22) reference and a distance limit of 500kb.

### Size simulation using the asthma GWAS data

Besides analyzing the MAGICS asthma GWAS data, we also conducted size simulations by permuting the disease status in the data. Logistic regression was considered where the dependent variable was the disease status (affected or unaffected) and the independent variables included a single SNP effect, gender, and the first principal component. The regression was applied to all the ~300,000 SNPs across the whole genome. Five thousand permutations were done by permuting the disease status among all the individuals, and then the model was refitted for each SNP. In the end of simulations, 5000 permutation *p*-values were obtained for each of the ~300,000 SNPs.

### Conflict of interest statement

The authors declare that the research was conducted in the absence of any commercial or financial relationships that could be construed as a potential conflict of interest.

## Resources

R functions have been made available for users' convenience to compute the eQTL weighted p-values in order to conduct weighted procedures such as Bonferroni, Holm's and Benjamini–Hochberg procedures. The functions can be found at http://www.hsph.harvard.edu/liming-liang/eqtl-weighted-gwas.

## Appendix

**Table A1:** **Published asthma associated SNPs in the GWAS catalog as of January 15, 2013**.

**Region**	**Chr**	**SNP**	**Context**	**Gene**	**eQTL**
1q21.3	1	rs4129267	Intron	*IL6R*	
1q23.1	1	rs1101999	Intron	*PYHIN1*	
1q21.3	1	rs4845783	Intergenic	*CRCT1*	
1q31.3	1	rs2786098	Intron	*DENND1B, CRB1*	
2q12.1	2	rs13408661	Intron	*IL1RL1, IL18R1*	
2q12.1	2	rs9807989	Intergenic	*IL18R1,IL1R1*	
2q12.1	2	rs3771180	Intron	*IL1RL1*	
2q12.1	2	rs3771166	Intron	*IL18R1*	Y
4q31.21	4	rs7686660	Intergenic	*LOC729675*	
4q31.21	4	rs3805236	Intron	*GAB1*	
5q31.3	5	rs6867913	Intergenic	*NDFIP1*	
5q31.1	5	rs11745587	Intron	*C5orf56*	Y
5q22.1	5	rs1837253	Intergenic	*TSLP*	
5q31.1	5	rs1295686	Intron	*IL13*	
5q31.1	5	rs2073643	Intron	*SLC22A5*	Y
5q31.1	5	rs2244012	Intron	*RAD50*	Y
5q12.1	5	rs1588265	Intron	*PDE4D*	
6p21.32	6	rs9268516	Intergenic	*BTNL2, HLA-DRA*	
6q27	6	rs6456042	Intergenic	*T*	
6p21.32	6	rs9500927	Intergenic	*HLA-DOA*	Y
6p21.32	6	rs9275698	Intergenic	*HLA-DQA2*	Y
6p21.32	6	rs7775228	Intergenic	*HLA-DQB1*	Y
6p21.32	6	rs3129890	Intergenic	*HLA-DRA*	
6p21.32	6	rs3117098	Intergenic	*BTNL2*	Y
6p21.32	6	rs204993	Intron	*PBX2*	Y
6p21.32	6	rs404860	Intron	*NOTCH4*	
6p21.32	6	rs3129943	Intron	*C6orf10*	Y
6p21.32	6	rs987870	Intron; nearGene-5	*HLA, DPB1*	Y
6p21.32	6	rs9273349	Intergenic	*HLA-DQ*	
8q24.11	8	rs3019885	Intron	*SLC30A8*	
9p21.1	9	rs10970976	Intron	*ACO1*	
9p24.1	9	rs2381416	Intergenic	*IL33*	
9p24.1	9	rs1342326	Intergenic	*IL33*	
10q21.1	10	rs7922491	Intron	*PRKG1*	
10p14	10	rs10508372	Intergenic	*LOC338591*	
11q13.5	11	rs7130588	Intergenic	*LRRC32*	
11q23.2	11	rs11214966	Intergenic	*C11orf71*	
12q13.2	12	rs1701704	Intergenic	*IKZF4*	Y
12q13.2	12	rs2069408	Intron	*CDK2*	Y
13q21.31	13	rs3119939	Intergenic	*PCDH20*	
15q22.2	15	rs11071559	Intron	*RORA*	
15q22.33	15	rs744910	Intron	*SMAD3*	
15q21.2	15	rs17525472	Intergenic	*SCG3*	Y
17q12	17	rs4794820	Intergenic	*ORMDL3*	Y
17q12	17	rs11078927	Intron	*GSDMB*	Y
17q12	17	rs6503525	Intergenic	*ORMDL3*	Y
17q21.1	17	rs3894194	Missense	*GSDMA*	Y
17q12	17	rs2305480	Missense	*GSDMB*	Y
17q12	17	rs7216389	Intron	*ORMDL3*	Y
19q13.42	19	rs16984547	Intron	*ZNF665*	
20p13	20	rs4815617	nearGene-5	*KIAA1271*	
22q12.3	22	rs2284033	Intron	*IL2RB*	

*“Y” in the eQTL column means the corresponding SNP is an eQTL SNP according to the eQTL database described in Materials and Methods. “nearGene-5” is an NCBI dbSNP function code, meaning that SNP is 5′ to and 2kb away from a gene*.

**Table A2:** **The significant SNPs identified by the unweighted method and the weighted methods (using general weight or binary weight) in the MAGICS analysis**.

**Rank**	**Unweighted method**			**General weight**			**Binary weight**		
	**Chr**	**SNP**	***p*-value**	**Chr**	**SNP**	***Q*_*g*_**	**Chr**	**SNP**	***Q*_*b*_**
1	17	rs3894194^*^	4.01E-09	17	rs3894194^*^	1.68E-09	17	rs3894194^*^	1.08E-09
2	17	rs8079416^*^	6.86E-09	17	rs8079416^*^	2.70E-09	17	rs8079416^*^	1.85E-09
3	17	rs4795408^*^	2.40E-08	17	rs4795408^*^	9.66E-09	17	rs4795408^*^	6.49E-09
4	17	rs3859192	3.58E-08	17	rs2290400^*^	1.75E-08	17	rs2290400^*^	2.00E-08
5	17	rs2290400^*^	7.39E-08	17	rs7216389^*^	1.76E-08	17	rs7216389^*^	2.02E-08
6	17	rs7216389^*^	7.48E-08	17	rs3859192	4.31E-08	17	rs3859192	5.25E-08
7				17	rs3902025^*^	1.05E-07	17	rs3902025^*^	7.44E-08
8				17	rs4795405^*^	1.07E-07	17	rs4795405^*^	9.83E-08
9				17	rs2305480^*^	1.40E-07	17	rs2305480^*^	1.54E-07

*Bonferroni's correction is used with an FWER level of 0.05*.

* *eQTL*.

**Table A3:** **The significant SNPs identified by the unweighted method and the weighted methods (using general weight or binary weight) in the MAGICS analysis**.

**Rank**	**Unweighted method**			**General weight**			**Binary weight**		
	**Chr**	**SNP**	***p*-value**	**Chr**	**SNP**	***Q*_*g*_**	**Chr**	**SNP**	***Q*_*b*_**
1	17	rs3894194^*^	4.01E-09	17	rs3894194^*^	1.68E-09	17	rs3894194^*^	1.08E-09
2	17	rs8079416^*^	6.86E-09	17	rs8079416^*^	2.70E-09	17	rs8079416^*^	1.85E-09
3	17	rs4795408^*^	2.40E-08	17	rs4795408^*^	9.66E-09	17	rs4795408^*^	6.49E-09
4	17	rs3859192	3.58E-08	17	rs2290400^*^	1.75E-08	17	rs2290400^*^	2.00E-08
5	17	rs2290400^*^	7.39E-08	17	rs7216389^*^	1.76E-08	17	rs7216389^*^	2.02E-08
6	17	rs7216389^*^	7.48E-08	17	rs3859192	4.31E-08	17	rs3859192	5.25E-08
7				17	rs3902025^*^	1.05E-07	17	rs3902025^*^	7.44E-08
8				17	rs4795405^*^	1.07E-07	17	rs4795405^*^	9.83E-08
9				17	rs2305480^*^	1.40E-07	17	rs2305480^*^	1.54E-07

*Holm's method is used with an FWER level of 0.05*.

* *eQTL*.

**Table A4:** **The positive results (SNPs) given by the unweighted method and the weighted methods (using general weight or binary weight) in the MAGICS analysis**.

**Rank**	**Unweighted method**			**General weight**			**Binary weight**		
	**Chr**	**SNP**	***p*-value**	**Chr**	**SNP**	***Q*_*g*_**	**Chr**	**SNP**	***Q*_*b*_**
1	17	rs3894194^*^	4.01E-09	17	rs3894194^*^	1.68E-09	17	rs3894194^*^	1.08E-09
2	17	rs8079416^*^	6.86E-09	17	rs8079416^*^	2.70E-09	17	rs8079416^*^	1.85E-09
3	17	rs4795408^*^	2.40E-08	17	rs4795408^*^	9.66E-09	17	rs4795408^*^	6.49E-09
4	17	rs3859192	3.58E-08	17	rs2290400^*^	1.75E-08	17	rs2290400^*^	2.00E-08
5	17	rs2290400^*^	7.39E-08	17	rs7216389^*^	1.76E-08	17	rs7216389^*^	2.02E-08
6	17	rs7216389^*^	7.48E-08	17	rs3859192	4.31E-08	17	rs3859192	5.25E-08
7	17	rs3902025^*^	2.75E-07	17	rs4795405^*^	1.07E-07	17	rs3902025^*^	7.44E-08
8	17	rs4795405^*^	3.64E-07	17	rs3902025^*^	1.05E-07	17	rs4795405^*^	9.83E-08
9	17	rs2305480^*^	5.68E-07	17	rs2305480^*^	1.40E-07	17	rs2305480^*^	1.54E-07
10	17	rs11557467	2.19E-06	17	rs8067378^*^	4.58E-07	17	rs8067378^*^	5.45E-07
11	17	rs8067378^*^	2.02E-06	17	rs9303277^*^	7.94E-07	17	rs9303277^*^	9.30E-07
12				17	rs1877031^*^	1.54E-06	17	rs1877031^*^	1.17E-06
13				17	rs907092^*^	1.56E-06	17	rs931992^*^	1.52E-06
14				17	rs931992^*^	1.96E-06	17	rs907092	1.68E-06
15				17	rs2941504^*^	2.68E-06	17	rs2941504^^*^^	2.06E-06
16				17	rs1565922^^*^^	2.55E-06	17	rs1565922^^*^^	1.97E-06
17				17	rs11557467	2.64E-06	10	rs11191325^^*^^	2.29E-06
18				10	rs11191325^^*^^	3.37E-06	17	rs11557467	3.22E-06
19							17	rs1007654^^*^^	3.11E-06

*The BH procedure is used with an FDR level of 0.05*.

* *eQTL*.

## References

[B1] Adzhubei I. A. Schmidt S. Peshkin L. Ramensky V. E. Gerasimova A. Bork P. (2010). A method and server for predicting damaging missense mutations. Nat. Methods 7, 248–249 10.1038/nmeth0410-248 20354512 PMC2855889

[B2] Anantharaman R. Andiappan A. K. Nilkanth P. P. Suri B. K. Wang D. Y. Chew F. T. (2011). Genome-wide association study identifies PERLD1 as asthma candidate gene. BMC Med. Genet. 12:170 10.1186/1471-2350-12-170 22188591 PMC3268734

[B3] Anderson C. A. Pettersson F. H. Clarke G. M. Cardon L. R. Morris A. P. Zondervan K. T. (2010). Data quality control in genetic case-control association studies. Nat. Protoc. 5, 1564–1573 10.1038/nprot.2010.116 21085122 PMC3025522

[B4] Benjamini Y. Hochberg Y. (1995). Controlling the false discovery rate: a practical and powerful approach to multiple testing. J. R. Stat. Soc. Ser. B 57, 289–300 10.2307/2346101

[B5] Bonferroni C. E. (1936). Teoria statistica delle classi e calcolo delle probabilità. Pubblicazioni del R Istituto Superiore di Scienze Economiche e Commerciali di Firenze 8, 3–62

[B6] Bouzigon E. Corda E. Aschard H. Dizier M. H. Boland A. Bousquet J. (2008). Effect of 17q21 variants and smoking exposure in early-onset asthma. N. Engl. J. Med. 359, 1985–1994 10.1056/NEJMoa0806604 18923164

[B7] Chu X. Pan C.-M. Zhao S.-X. Liang J. Gao G.-Q. Zhang X.-M. (2011). A genome-wide association study identifies two new risk loci for Graves' disease. Nat. Genet. 43, 897–901 10.1038/ng.898 21841780

[B8] Cookson W. Liang L. Abecasis G. Moffatt M. Lathrop M. (2009). Mapping complex disease traits with global gene expression. Nat. Rev. Genet. 10, 184–194 10.1038/nrg2537 19223927 PMC4550035

[B9] Cookson W. (2004). The immunogenetics of asthma and eczema: a new focus on the epithelium. Nat. Rev. Immunol. 4, 978–988 10.1038/nri1500 15573132

[B10] Dimas A. S. Deutsch S. Stranger B. E. Montgomery S. B. Borel C. Attar-Cohen H. (2009). Common regulatory variation impacts gene expression in a cell type-dependent manner. Science 325, 1246–1250 10.1126/science.1174148 19644074 PMC2867218

[B11] Ding J. Gudjonsson J. E. Liang L. Stuart P. E. Li Y. Chen W. (2010). Gene expression in skin and lymphoblastoid cells: refined statistical method reveals extensive overlap in cis-eQTL signals. Am. J. Hum. Genet. 87, 779–789 10.1016/j.ajhg.2010.10.024 21129726 PMC2997368

[B12] Dixon A. L. Liang L. Moffatt M. F. Chen W. Heath S. Wong K. C. C. (2007). A genome-wide association study of global gene expression. Nat. Genet. 39, 1202–1207 10.1038/ng2109 17873877

[B15] Genovese C. R. Roeder K. Wasserman L. (2006). False discovery control with p-value weighting. Biometrika 93, 509–524 10.1198/jasa.2010.tm09329 21931466 PMC3175141

[B16] Heid I. M. Jackson A. U. Randall J. C. Winkler T. W. Qi L. Steinthorsdottir V. (2010). Meta-analysis identifies 13 new loci associated with waist-hip ratio and reveals sexual dimorphism in the genetic basis of fat distribution. Nat. Genet. 42, 949–960 10.1038/ng.685 20935629 PMC3000924

[B17] Hoggart C. J. Clark T. G. De Iorio M. Whittaker J. C. Balding D. J. (2008). Genome-wide significance for dense SNP and resequencing data. Genet. Epidemiol. 32, 179–185 10.1002/gepi.20292 18200594

[B18] Holm S. (1979). A simple sequentially rejective multiple test procedure. Scand. J. Stat. 6, 65–70 10.2307/4615733

[B19] Hosack D. A. Dennis G. Jr. Sherman B. T. Lane H. C. Lempicki R. A. (2003). Identifying biological themes within lists of genes with EASE. Genome Biol. 4:R70 10.1186/gb-2003-4-10-r70 14519205 PMC328459

[B20] Hsu Y.-H. Zillikens M. C. Wilson S. G. Farber C. R. Demissie S. Soranzo N. (2010). An integration of genome-wide association study and gene expression profiling to prioritize the discovery of novel susceptibility Loci for osteoporosis-related traits. PLoS Genet. 6:e1000977 10.1371/journal.pgen.1000977 20548944 PMC2883588

[B21] Johnson A. D. Handsaker R. E. Puilt S. Nizzari M. M. O'Donnell C. J. de Bakker P. I. W. (2008). SNAP: a web-based tool for identification and annotation of proxy SNPs using HapMap. Bioinformatics 24, 2938–2939 10.1093/bioinformatics/btn564 18974171 PMC2720775

[B22] Lango Allen H. Estrada K. Lettre G. Berndt S. I. Weedon M. N. Rivadeneira F. (2010). Hundreds of variants clustered in genomic loci and biological pathways affect human height. Nature 467, 832–838 10.1038/nature09410 20881960 PMC2955183

[B23] Li M. X. Yeung J. M. Y. Cherny S. S. Sham P. C. (2012). Evaluating the effective numbers of independent tests and significant p-value thresholds in commercial genotyping arrays and public imputation reference datasets. Hum. Genet. 131, 747–756 10.1007/s00439-011-1118-2 22143225 PMC3325408

[B24] Liang L. Morar N. Dixon A. L. Lathrop G. M. Abecasis G. R. Moffatt M. F. (2013). A cross-platform catalogue of 14, 177 expression quantitative trait loci derived from lymphoblastoid cell lines. Genome Res. 23, 716–726 10.1101/gr.142521.112 23345460 PMC3613588

[B25] Moffatt M. F. Kabesch M. Liang L. Dixon A. L. Strachan D. Heath S. (2007). Genetic variants regulating ORMDL3 expression contribute to the risk of childhood asthma. Nature 448, 470–473 10.1038/nature06014 17611496

[B26] Moffatt M. F. Gut I. G. Demenais F. Strachan D. P. Bouzigon E. Heath S. (2010). A large-scale, consortium-based genomewide association study of asthma. N. Engl. J. Med. 363, 1211–1221 10.1056/NEJMoa0906312 20860503 PMC4260321

[B27] Nica A. C. Parts L. Glass D. Nisbet J. Barrett A. Sekowska M. (2011). The architecture of gene regulatory variation across multiple human tissues: the MuTHER study. PLoS Genet. 7:e1002003 10.1371/journal.pgen.1002003 21304890 PMC3033383

[B28] Nicolae D. L. Gamazon E. Zhang W. Duan S. Dolan M. E. Cox N. J. (2010). Trait-associated SNPs are more likely to be eQTLs: annotation to enhance discovery from GWAS. PLoS Genet. 6:e1000888 10.1371/journal.pgen.1000888 20369019 PMC2848547

[B29] Patterson N. Price A. L. Reich D. (2006). Population structure and eigenanalysis. PLoS Genet. 2:e190 10.1371/journal.pgen.0020190 17194218 PMC1713260

[B30] Price A. L. Patterson N. J. Plenge R. M. Weinblatt M. E. Shadick N. A. Reich D. (2006). Principal components analysis corrects for stratification in genome-wide association studies. Nat. Genet. 38, 904–909 10.1038/ng1847 16862161

[B31] Purcell S. Neale B. Todd-Brown K. Thomas L. Ferreira M. A. R. Bender D. (2007). PLINK: a tool set for whole-genome association and population-based linkage analyses. Am. J. Hum. Genet. 81, 559–575 10.1086/519795 17701901 PMC1950838

[B32] Roeder K. Wasserman L. (2009). Genome-wide significance levels and weighted hypothesis testing. Stat. Sci. 24, 398–413 10.1214/09-STS289 20711421 PMC2920568

[B33] Roeder K. Bacanu S.-A. Wasserman L. Devlin B. (2006). Using linkage genome scans to improve power of association in genome scans. Am. J. Hum. Genet. 78, 243–252 10.1086/500026 16400608 PMC1380233

[B34] Roeder K. Devlin B. Wasserman L. (2007). Improving power in genome-wide association studies: weights tip the scale. Genet. Epidemiol. 31, 741–747 10.1002/gepi.20237 17549760

[B35] Saccone S. F. Hinrichs A. L. Saccone N. L. Chase G. A. Konvicka K. Madden P. (2007). Cholinergic nicotinic receptor genes implicated in a nicotine dependence association study targeting 348 candidate genes with 3713 SNPs. Hum. Mol. Genet. 16, 36–49 10.1093/hmg/ddl438 17135278 PMC2270437

[B36] Speliotes E. K. Willer C. J. Berndt S. I. Monda K. L. Thorleifsson G. Jackson A. U. (2010). Association analyses of 249, 796 individuals reveal 18 new loci associated with body mass index. Nat. Genet. 42, 937–948 10.1038/ng.686 20935630 PMC3014648

[B37] Stephens M. Balding D. (2009). Bayesian statistical methods for genetic association studies. Nat. Rev. Genet. 10, 681–690 10.1038/nrg2615 19763151

[B38] Storey J. D. Tibshirani R. (2003). Statistical significance for genomewide studies. Proc. Natl. Acad. Sci. U.S.A. 100, 9440–9445 10.1073/pnas.1530509100 12883005 PMC170937

[B39] Stranger B. E. Forrest M. S. Clark A. G. Minichiello M. J. Deutsch S. Lyle R. (2005). Genome-wide associations of gene expression variation in humans. PLoS Genet. 1:e78 10.1371/journal.pgen.0010078 16362079 PMC1315281

[B40] Stranger B. E. Nica A. C. Forrest M. S. Dimas A. Bird C. P. Beazley C. (2007). Population genomics of human gene expression. Nat. Genet. 39, 1217–1224 10.1038/ng2142 17873874 PMC2683249

[B41] Stranger B. E. Montgomery S. B. Dimas A. S. Parts L. Stegle O. Ingle C. E. (2012). Patterns of cis regulatory variation in diverse human populations. PLoS Genet. 8:e1002639 10.1371/journal.pgen.1002639 22532805 PMC3330104

[B42] Teslovich T. M. Musunuru K. Smith A. V. Edmondson A. C. Stylianou I. M. Koseki M. (2010). Biological, clinical and population relevance of 95 loci for blood lipids. Nature 466, 707–713 10.1038/nature09270 20686565 PMC3039276

[B43] Wang K. Li M. Bucan M. (2007). Pathway-based approaches for analysis of genomewide association studies. Am. J. Hum. Genet. 81, 1278–1283 10.1086/522374 17966091 PMC2276352

[B44] Wellcome Trust Case Control Consortium. (2007). Genome-wide association study of 14, 000 cases of seven common diseases and 3, 000 shared controls. Nature 447, 661–678 10.1038/nature05911 17554300 PMC2719288

[B45] Wu C. Miao X. Huang L. Che X. Jiang G. Yu D. (2012). Genome-wide association study identifies five loci associated with susceptibility to pancreatic cancer in Chinese populations. Nat. Genet. 44, 62–66 10.1038/ng.1020 22158540

[B46] Xiong Q. Ancona N. Hauser E. R. Mukherjee S. Furey T. S. (2012). Integrating genetic and gene expression evidence into genome-wide association analysis of gene sets. Genome Res. 22, 386–397 10.1101/gr.124370.111 21940837 PMC3266045

[B47] Yang T.-P. Beazley C. Montgomery S. B. Dimas A. S. Gutierrez-Arcelus M. Stranger B. E. (2010). Genevar: a database and Java application for the analysis and visualization of SNP-gene associations in eQTL studies. Bioinformatics 26, 2474–2476 10.1093/bioinformatics/btq452 20702402 PMC2944204

[B48] Zhang M. Liang L. Morar N. Dixon A. L. Lathrop G. M. Ding J. (2012). Integrating pathway analysis and genetics of gene expression for genome-wide association study of basal cell carcinoma. Hum. Genet. 131, 615–623 10.1007/s00439-011-1107-5 22006220 PMC3303995

[B49] Zhong H. Yang X. Kaplan L. M. Molony C. Schadt E. E. (2010). Integrating pathway analysis and genetics of gene expression for genome-wide association studies. Am. J. Hum. Genet. 86, 581–591 10.1016/j.ajhg.2010.02.020 20346437 PMC2850442

